# Ultraviolet- attenuated cercariae of *Schistosoma japonicum* fail to effectively induce a Th1 response in spite of up-regulating expression of cytotoxicity-related genes in C57BL/6 mice^☆^

**DOI:** 10.1016/S1674-8301(10)60039-5

**Published:** 2010-07

**Authors:** Meijuan Zhang, Fang Tian, Yanan Gao, Minjun Ji, Guanling Wu

**Affiliations:** a Department of Pathogen Biology and Immunology, Nanjing Medical University, Nanjing 210029, Jiangsu Province, China; b Department of Clinical Laboratory, the first Affliated Hospital of Nanjing Medical University, Nanjing 210029, Jiangsu Province, China; c Jiangsu Province Key Laboratory of Modern Pathogen Biology, Nanjing 210029, Jiangsu Province, China

**Keywords:** *Schistosoma japonicum*, ultraviolet-attenuated cercariae, Th1 response, cytotoxicity-related genes, C57BL/6 mice

## Abstract

**Objective:**

To better understand the reason that *Schistosoma japonicum* (*S. japonicum*) ultraviolet (UV)-radiated cercariae could not induce high level of protection in C57BL/6 mice.

**Methods:**

Microarray technology was performed to investigate the gene transcription profile in skin draining lymph nodes (sdLNs) at 1 w after exposure to attenuated cercariae (AC) or normal cercariae (NC) of *S. japonicum* in C57BL/6 mice. The expressions of some representative genes were further confirmed by real-time PCR. Subsequently, the expressions of Th1/Th2 cytokine genes, cytotoxicity-related genes, as well as co-stimulator genes in spleens from AC-vaccinated and NC-infected mice were analyzed by real-time PCR at w 3 and 6 post-exposure.

**Results:**

The gene expressions of Th1 cytokines, including interferon-γ (IFN-γ), interleukin (IL)-12 and tumor necrosis factor-α (TNF-α) in the sdLNs were significantly lower in AC-vaccinated mice than in NC-infected mice. Furthermore, the gene expressions of Th1- and Th2- cytokines, including IFN-γ, IL-12, TNF-α, IL-4 and IL-10, in the spleens from AC-vaccinated mice showed little changes at w 3 and 6 post-vaccination. In addition, cytotoxicity-related molecules including granzyme A, granzyme B, granzyme K, perforin 1 and Fas L were up-regulated from the early stage of vaccination, and peaked at the 3^rd^ w after vaccination with UV-AC.

**Conclusion:**

UV-AC of *S. japonicum* could not effectively induce a Th1 response in C57BL/6 mice, which may be an explanation for the low protection against parasite challenge, and the role played by up-regulated expression of cytotoxicity-related genes in mice needs to be further investigated.

## INTRODUCTION

Schistosomiasis japonica, caused by the trematode *Schistosoma japonicum* (*S. japonicum*), is prevalent mainly in China, Philippines and Indonesia, and causes a significant public health problem in terms of morbidity and mortality[1]. Although much effort has been devoted to the development of vaccines against schistosomiasis, the lack of a thorough understanding of the protective immunity has hampered the emergence of effective vaccines against schistosomes, especially *S. japonicum*.

Attenuation of invasive cercariae with ionizing radiation [γ, X-rays, or ultraviolet(UV)], based upon the development of successful viral and bacterial vaccines in the early 20^th^ century[2]–[5], has been recognized as an appealing vaccination strategy. Radiation-attenuated cercariae (AC) have been proven to defend effectively against *S. mansoni*. A single exposure to attenuated cercariae is sufficient to achieve protection of 60%-80% in mice[6]. This resistance developed in mice is considered to be the result of a cell-mediated immune response that is predominantly dependent upon CD4^+^ T lymphocytes, specifically the Th1 subset[1],[7]. Also, *S. mansoni* radiation-AC could induce protection of 60%-90% in non-human primates and some domestic animals, in which an antibody response is a fundamental contributor to the acquired resistance against challenge[8]–[11].

In some studies of *S. japonicum*, UV-AC could also produce high level protection in artiodactyls, 92% in pigs[12],[13] and 89.1% in cattle[14], against *S. japonicum* infections. However, most of the studies from different laboratories have come to the conclusion that protection in mice induced by attenuated *S. japonicum* cercariae is unstable and relatively low. Gui *et al*[15] and our group[16] reported that UV-AC produced protection of 1.30%-22.70% and 2.27%-38.67%, respectively against *S. japonicum* challenge in C57BL/6 mice. These significant differences between domestic animals and mice in the protective efficiency of vaccination with attenuated *S. japonicum* cercariae suggest that the mouse might not be a good model to study vaccines against schistosomiasis japonica. However, the mechanisms underlying the lack of a protective response in mice are worth studying. By investigating various immunological events concomitant to low level protection and comparing them to protective responses, researchers can infer possible mechanisms involved in the protection in some animal models.

Since the importance of skin-draining lymph nodes (sdLNs) has been well established in the induction of protection, we first observed the gene transcription profile in sdLNs at w 1 after exposure to UV-AC or normal cercariae (NC) of *S. japonicum* in C57BL/6 mice. After vaccination with AC or infection with NC *in vivo*, the cell-mediated responses in spleens were dynamically investigated and analyzed until 6 w post-exposure.

## MATERIALS AND METHODS

### Mice and parasites

Female C57BL/6 mice of 8 to 10-week-old were purchased from the Model Animal Research Center of Nanjing University (China). *S. japonicum* (a Chinese mainland strain) cercariae were maintained in *Oncomelania hupensis* snails as the intermediate host, and were purchased from Jiangsu Institute of Parasitic Disease (China). All experiments were undertaken with the approval of Nanjing Medical University Animal Ethics Committee.

### Infection or vaccination of mice and sample collection

Freshly shed *S. japonicum* cercariae were attenuated by UV radiation using a portable UV lamp (type N16; Konrad Benda, Laborgerate, D-6908 Wiesloch, FRG) at 254 nm with an intensity of 400 µw/cm^2^ for 1 min. Mice were percutaneously infected or vaccinated with 20 NC or 300 UV-AC through their shaved abdomen for 20 min by the cover glass method, respectively. At w 1 after infection or vaccination, 5 mice from each group were sacrificed and their sdLNs, including axillary and inguinal lymph nodes were collected, homogenated and stored in TRIzol reagent. At w 3 and 6 post-infection or vaccination, the mice were sacrificed and spleens were aseptically harvested and prepared for mononuclear cells, which were then stored in TRIzol reagent for gene expression analysis.

### Analysis of gene expression profile

#### Total RNA extraction and Affymetrix genechip protocols

Gene expression profiles of the sdLNs collected at one week after vaccination with AC or infection with NC were performed using microarray analysis. First, total RNA of 5 samples from each group was extracted using TRIzol reagent (Invitrogen Life Technologies, USA) and pooled in identical quantities, followed by purification with RNeasy kit (QIAGEN, USA). cDNA was generated using the One-Cycle Target Labeling and Control Reagents (Affymetrix, USA), and cRNA was made by GeneChip^®^ IVT Labeling Kit (Affymetrix). Biotin-labeled, fragmented (200 nt or less) cRNA was hybridized for 16 h at 45°C to Mouse Genome 430 2.0 arrays (Affymetrix) by the Microarray Facility. The arrays were washed and stained, and then read by GeneChip^®^ Scanner 3000 (Affymetrix). The fluorescence signal was excited at 570 nm, and data were collected on a confocal scanner at 3 µm resolution.

#### Oligonucleotide array data analysis

Data analysis was performed by GeneChip Operating Software 1.4. Initial absolute analyses for gene expression were performed without scaling while subsequent comparison analysis files were created by scaling all data sets to a uniform value (so-called Target Signal, 500) to normalize all probe sets. Pairwise comparison between AC-vaccinated and NC-infected samples was carried out. Each probe set in the microarray of an AC-vaccinated sample was compared with its counterpart in the microarray of the NC-infected sample, and the *P*-value of the difference was calculated by the Wilcoxon's signed-rank test.

#### Quantitative analysis of mRNA transcription by real-time PCR

Five samples in each group were collected at w 1 (sdLNs), 3 and 6 (spleen) after vaccination or infection and were further analyzed by real-time PCR. Three and one half micrograms of total RNA of each sample were reverse transcribed by MMLV Reverse Transcriptase (Epicentre, Germany) using an oligo(dT)18 primer (Invitrogen, USA). Afterwards, cDNAs were amplified with specific primers for each target gene using the ABI 7900 real-time PCR system (ABI, USA). Reactions were performed using 2 µL of cDNA in a 10 µL reaction volume and the following thermal cycle profile: 10 min of denaturation at 95 °C, 40 cycles of 15 s denaturation at 95 °C and then 60 s of extension at 60 °C. Primers specific for Th1/Th2 cytokine genes IFN-γ(*ifng*), IL-12 (*il12*), TNF-α (*tnfa*), IL-4 (*il4*), IL-10 (*il10*), and cytotoxicity- related genes including granzyme A (*gzma*), granzyme B (*gzmb*), granzyme K (*gzmk*), perforin 1 (*pfr1*), Fas L (*fasl*), as well as co-stimulator genes CD40 (*cd40*), CD86 (*cd86*) are shown in ***Table 1***. PCR amplification of *β*-*actin* was performed to allow normalization between samples.

**Table 1:** Primers and annealing temperatures used for the amplification of each target gene

*Gene*	Primer (5′→3′)	Annealing temperature (°C)	Product size(bp)
*β-actin*	F:5′CCTCTATGCCAACACAGTGC3′ R:5′GTACTCCTGCTTGCTGATCC3′	59	211
*ifng*	F:5′AGCAACAACATAAGCGTCAT3′ R:5′CCTCAAACTTGGCAATACTC3′	59	100
*il12*	F:5′TGATGATGACCCTGTGCCTT3′ R:5′CTGCTGATGGTTGTGATTCTG A3′	59	104
*tnfa*	F:5′GAGTCCGGGCAGGTCTACTTT3′ R:5′CAGGTCACTGTCCCAGCATCT3′	59	235
*il4*	F:5′CATCCTGCTCTTCTTTCTCG3′ R:5′CCTTCTCCTGTGACCTCGTT3′	59	105
*il10*	F:5′CAACATACTGCTAACCGACTC 3′ R:5′CATTCATGGCCTTGTAG AC AC3′	59	293
*gzma*	F:5′TGTGAAACCAGGAACCAGATG3′ R:5′GGTGATGCCTCGC AAA ATA3′	59	256
*gzmb*	F: 5′TGCTCTGATT ACCC ATCGTCC3′ R:5′GCCAGTCTTTGCAGTCCTTTATT3′	59	89
*gzmk*	F:5′CCCACTGCTACTCTTGGTTTC3′ R:5′GGCATTTGGTCCCATCTCTA3′	59	252
*prf1*	F:5′CAATGGCAAGTATGTGGTGGT3′ R:5′CAGTGAGATGGTTTCCCGAGT3′	59	139
*fasl*	F:5′GGTTCTGGTGGCTCTGGTT3′ R:5′ACTTTAAGGCTTTGGTTGGTG3′	59	105
*cd40*	F:5′CTGGTCTGAACCCTGGAACT3′ R:5′GGCTCTGTCTTGGCTCATCTC3′	59	113
*cd86*	F:5′TTCATTCCCGGATGGTGTG3′ R:5′GGCTGATTCGGCTTCTTGTG3′	59	204

### Statistical analysis

Results were expressed as mean±SD. The statistical significance of mean differences between two groups was calculated by an unpaired Student's *t*-test using SPSS software (Version 16.0, SPSS Inc., USA). Significant values were indicated as follows: *P < 0.05, **P < 0.01.

## RESULTS

### UV-irradiated *S. japonicum* cercariae produced low transcription levels of Th1-cytokine genes in sdLNs of C57BL/6 mice after 1w after vaccination.

**Fig. 1:**
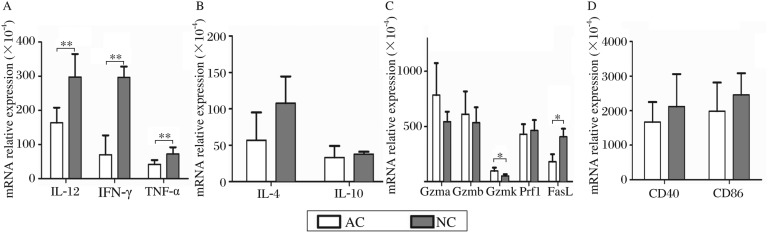
The transcription levels of Th1- and Th2- cytokines, cytotoxicity- related genes as well as co-stimulators in sdLNs from AC-vaccinated and NC-infected mice at w 1 after exposure (*n* = 5). The expression level for each gene (A: IL-12, LFN-γ and TNF-α. B: IL-4 and IL-10. C: Gzma, Gzmb, Gzmk, Prf1 and FasL. D: CD40 and CD86) was normalized to β-actin, and the difference of each gene expression level between AC and NC groups was compared (*P < 0.05, **P < 0.01)

We first investigated the early immune-related gene expression profile in the sdLNs after 1w post-exposure in the AC-vaccinated and NC-infected C57BL/6 mice by microarray analysis. The transcription level of some representative genes was further confirmed by real-time PCR. As depicted in ***Table 2*** (signal intensities from microarray) and ***Fig. 1*** (mRNA transcription levels obtained from real-time PCR), in the AC-vaccinated group at one week post-exposure, the mRNA levels of Th1 cytokines including *i112*, *ifng* and *tnfa* were significantly lower than those in the NC-infected group. However, the results of real-time PCR indicated that little difference was observed in the expression of Th2-cytokine genes including *il4* and *il10* between the AC-vaccinated and NC-infected groups. In addition, the expressions of co-stimulators (*cd40* and *cd86*) in AC-vaccinated mice were slightly lower than those in NC-infected mice, but the differences were not significant. Unlike the attenuated Th1 response, we unexpectedly found that some cytotoxicity-related genes including *gzma*, *gzmb* and *gzmk* exhibited higher transcription levels in the AC-vaccinated group from an early stage, especially for *gzmk* (*P* < 0.05 showed by real-time PCR). However, *fasl* expression was relatively low in AC-vaccinated mice.

**Table 2:** Signal intensities of Th1-and Th2- cytokines, cytotoxicity-related genes, as well as co-stimulators from microarray analysis in sdLNs from vaccinated and infected mice at w 1 after 1 w of exposure.

GenBank No.	Gene name	Signal intensity	
		AC-vaccinated sdLNs	NC-vaccinated sdLNs
16159	*Il12*	369.1	465.9
15978	*ifng*	134.3	182.8
21926	*tnfa*	41.2	241.0
16153	*il10*	80.3	122.9
16189	*il4*	104.9	47.7
14938	*gzma*	6685.4	4777.8
14939	*gzmb*	3150.0	1664.9
14945	*gzmk*	1185.8	539.7
18646	*prfl*	547.8	650.6
14103	*fasl*	204.3	214.3
21939	*cd40*	1857.3	1855.8
12524	*cd86*	1256.5	1698.1

### UV-irradiated cercariae failed to induce the CD4 response in spleens at w 3 and 6 after vaccination.

**Fig. 2:**
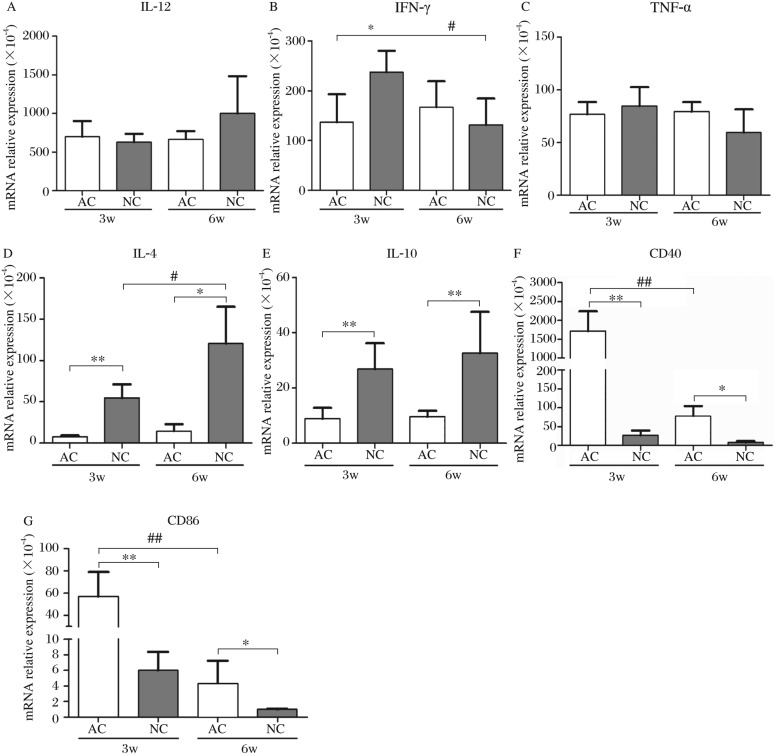
The transcription levels of Th1-and Th2- cytokines, co-stimulators in spleens from AC-vaccinated and NC-infected mice at w 3 and 6 after exposure, assessed by real-time PCR (*n* = 5). Expression levels for each gene (A: IL-12. B: LFN-γ. C: TNF-α. D: IL-4. E: IL-10. F: CD40. G: CD86) were normalized to β-actin. The difference of each gene's level between AC and NC groups was compared, and significant values were indicated as follows: *P < 0.05, **P < 0.01; the diffence of each gene's level between w 3 and 6 respectively in AC and NC groups was compared also, and significant values were indicated as follows: ^#^*P* < 0.05, ^##^*P* < 0.01.

To further focus on the dynamics of the CD4 response in mice induced by AC vaccination and NC infection, the mRNA transcription levels of Th1 and Th2 cytokines, and co-stimulators in splenocytes at w 3 and 6 after exposure were analyzed by real-time PCR. As shown in ***Fig. 2***, *il12* and *tnfa* did not show any increased expression in the 300 cercariae AC-vaccinated mice compared with those in 20 cercariae NC-infected mice during this period. Furthermore, *ifng* expression by the AC vaccinated mice was still lower than that in the NC-infected mice at the 3^rd^ w post-exposure. In addition, both *il4* and *il10* expression were kept at extremely low levels in AC-vaccinated mice without any eggs deposited in the tissue. Accordingly, with the progress of infection, the levels of *il4* increased quickly, especially at w 6 post-infection when a large number of eggs appeared, and *il10* expression showed a slight elevation. We also found that both *cd40* and *cd86* expressions were significantly induced at w 3 after vaccination with AC, and decreased at w 6. At the same time, these two co-stimulators showed much higher transcription in AC-vaccinated mice than in NC-infected mice.

### UV-irradiated cercariae resulted in significant up-regulation of cytotoxicity-related genes in spleens, especially at w 3 post-vaccination.

**Fig. 3:**
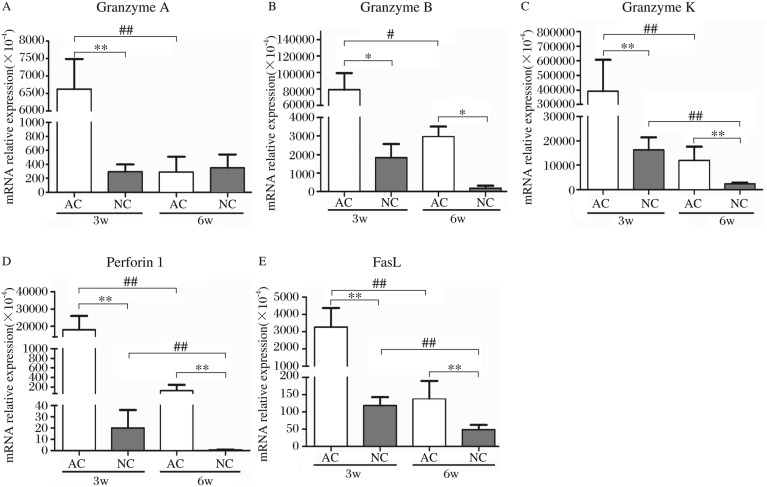
The transcription levels of cytotoxicity-related genes in spleens from AC-vaccinated and NC-infected mice at w 3 and 6 after exposure (*n* = 5). The expression level of each gene was normalized to β-actin. The difference of each gene's level between AC and NC groups was compared, and significant values were indicated as follows: *P < 0.05, **P < 0.01; the diffence of each gene's level of between w 3 and 6 respectively in AC and NC groups was compared also, and significant values were indicated as follows: ^#^*P* < 0.05, ^##^*P* < 0.01.

Since cytotoxicity-related genes in the sdLNs could be activated immediately with exposure to AC, we further observed the transcription levels of these genes, including granzyme A, granzyme B, granzyme K, perforin 1 and FasL, in spleens after 3 and 6 w of vaccination. As shown in ***Fig. 3***, after 3 w of AC vaccination, the mRNA expressions of *gzma, gzmb, gzmk, prf1* and *fasl* significantly increased, far exceeding the equivalent values in NC-infected mice. With the passage of time, the levels of these genes induced by AC vaccination were markedly decreased. On the other hand, the expressions of those cytotoxicity-related genes in NC-infected mice remained relatively low, and even achieved the lowest level at w 6 post-infection, with the exception of *gzma* expression.

## DISCUSSION

In our previous studies on *S. japonicum*[16], although different UV-radiation intensities (300, 400, 500 µw/cm^2^), different vaccination regimes and different mouse strains including C57BL/6, DBA, Kunming (our unpublished data) were used to evaluate the protective efficiency induced by AC, we did not obtain optimal vaccination conditions. *S. japonicum* UV-radiated cercariae produced unstable and low level of protection in mice. Based on these findings, in the present study we further analyzed the immune-related gene expression in the sdLNs and the spleens from the C57BL/6 mice vaccinated with UV-irradiated cercariae or infected with NC of *S. japonicum*, to help us better understand the possible protective mechanisms against schistosomiasis japonica by comparative analysis with studies on *S. mansoni*.

The sdLNs are important sites where the antigen is processed and presented to the effector cells to initiate the immune response[7],[17]. After 1 w of vaccination, our data obtained from microarray and real-time PCR showed that the CD4^+^ Th response, especially Th1 response, in sdLNs could not be activated by attenuating *S. japonicum* cercariae in this mouse model, as evidenced by low expressions of IFN-γ, IL-12 and TNF-α. Meanwhile, the expressions of co-stimulators, especially CD40 were inhibited slightly. These results were quite inconsistent with the studies on radiated *S. mansoni* cercariae. Lu *et al*[18] found that T cell proliferation in the sdLNs elicited by UV-attenuated *S. mansoni* cercariae was stronger and more sustained than that induced by NC. Several studies showed that vaccination with irradiated cercariae of *S. mansoni*, preferentially induced the accumulation of IFN-γ producing T cells in the skin and sdLNs of mice[19]. Th1 cells generated in the sdLNs and then enter the circulation, and a proportion of these are schistosome-specific, as they can provoke delayed type hypersensitivity reactions following challenge with parasite antigen[20]. IL-12 was also a critical component of this Th1-mediated protection[21],[22]. In addition, the number of dendritic cells (DC) transfered into the sdLNs after vaccination with attenuated *S. manosi* cercariae was more than that induced by NC[23]. CD40/CD154 interactions were required for the optimal maturation of skin-derived antigen-presenting cells (APCs) and the induction of *S. mansoni* antigen-specific IFN-γ[24]. These data suggest that the predominant Th1 response in the skin and sdLNs is central to the induction of protective immunity by attenuated *S. mansoni* cercariae. However, in the present study, UV-irradiated cercariae of *S. japonicum* only produced a low level Th1 response in the sdLNs of C57BL/6 mice. Even by 6 w after vaccination, irradiated cercariae failed to provoke an effective Th1 response in mice. An attenuated CD4^+^ T cell response, especially a Th1 response, might to some extent result in the lack of memory T cell differentiation, which would not form a favorable environment against challenge.

Although UV-AC could not effectively promote CD4^+^ T cell function in C57BL/6 mice, they did enhance the transcription levels of some cytotoxicity-related genes, including granzyme A, B, K, perforin and FasL from the early stage of vaccination, reaching a peak at w 3 post-vaccination. This might indicate that CD8^+^ T cells could be activated directly by AC when the protection derived from CD4^+^ T cells was limited. Accordingly, high expressions of co-stimulators were also observed at w 3 after vaccination, which might suggest that irradiated cercariae could stimulate the APCs to activate the CD8^+^ response by cross-presentation[25] or by some other unknown mechanisms. Kumar *et al*[7] also demonstrated a significant increase in the CD8^+^ T cells in the skin and its sdLNs of mice vaccinated with γ-irradiated *S. mansoni* cercariae[7], but the exact role of these CD8^+^ cells in the protection against such a multicellular pathogen, schistosome, was not clear. In contrast, the expressions of those cytotoxic genes in normal cercariae-infected mice remained at a low level. Thus, although a strong CD8^+^ T cell response was induced by attenuated *S. japonicum* cercariae, this effect seemed to be insufficient to produce a powerful resistance against *S. japonicum* challenge in mice.

In conclusion, the CD4^+^ T cell response, especially Th1 response in the early stage of the vaccination is more important than CD8^+^ T cells for the induction of protective immunity to *S. japonicum* infection. The mutual coordination between CD4^+^ and CD8^+^ T cell functions might achieve the perfect protection. Furthermore, for UV-AC of *S. japonicum*, the mouse might not be an ideal model for studying the mechanisms of protection against *S. japonicum*.

## References

[1] McManus DP, Loukas A (2008). Current status of vaccines for schistosomiasis. Clin Microbiol Rev.

[2] Bickle QD, Dobinson T, James ER (1979). The effects of gamma-irradiation on migration and survival of *Schistosoma mansoni* schistosomula in mice. Parasitol.

[3] Hsu SY, Hsu HF, Burmeister LF (1981). *Schistosoma mansoni*: vaccination of mice with highly x-irradiated cercariae. Exp Parasitol.

[4] Minard P, Dean DA, Jacobson RH, Vannier WE, Murrell KD (1978). Immunization of mice with cobalt-60 irradiated Schistosoma mansoni cercariae. Am J Trop Med Hyg.

[5] Murrell KD, Clark S, Dean DA, Vannier WE (1979). Influence of mouse strain on induction of resistance with irradiated *Schistosoma mansoni* cercariae. J Parasitol.

[6] Anderson S, Coulson PS, Ljubojevic S, Mountford AP, Wilson RA (1999). The radiation-attenuated schistosome vaccine induces high levels of protective immunity in the absence of B cells. Immunol.

[7] Kumar P, Ramaswamy K (1999). Vaccination with irradiated cercariae of *Schistosoma mansoni* preferentially induced the accumulation of interferon-gamma producing T cells in the skin and skin draining lymph nodes of mice. Parasitol Int.

[8] Bickle QD (2009). Radiation-attenuated schistosome vaccination--a brief historical perspective. Parasitol.

[9] Coulson PS, Kariuki TM (2006). Schistosome vaccine testing: lessons from the baboon model. Mem Inst Oswaldo Cruz.

[10] Kariuki TM, Farah IO, Yole DS (2004). Parameters of the attenuated schistosome vaccine evaluated in the olive baboon. Infect Immun.

[11] Yole DS (1996). Protective immunity to *Schistosoma mansoni* induced in the olive baboon Papio anubis by the irradiated cercaria vaccine. Parasitol.

[12] Bickle QD (2001). Comparison of the vaccine efficacy of gamma-irradiated *Schistosoma japonicum* cercariae with the defined antigen Sj62 (IrV-5) in pigs. Vet Parasitol.

[13] Shi YE, Jiang CF, Han JJ, Li YL, Ruppel A (1993). Immunization of pigs against infection with *Schistosoma japonicum* using ultraviolet-attenuated cercariae. Parasitol.

[14] Hsu SY, Xu ST, He YX, Shi FH, Shen W, Hsu HF (1984). Vaccination of bovines against schistosomiasis japonica with highly irradiated schistosomula in China. Am J Trop Med Hyg.

[15] Gui M, Wales A, Jones JT (1993). Vaccination of mice with radiation attenuated larvae of *Schitosoma japonicum* or *S. mansoni*. Mitt Osterr Ges Tropenmed, Parasitol.

[16] Hu SY, Zhang MJ, Guo CQ, Shan H, Zhang SH (2006). Study on the protection of C57BL/ 6 mice vaccinated with UV attenuated *Schistosoma japonicum* cercariae. J Pathogen Biology (in chinese).

[17] Mountford AP, Wilson RA (1990). *Schistosoma mansoni*: the effect of regional lymphadenectomy on the level of protection induced in mice by radiation-attenuated cercariae. Exp Parasitol.

[18] Lu FL, Gui M, Filsinger S, Hansch GM, Ruppel A (1995). Comparative phenotypic analysis of lymph node cells in mice after infection or vaccination with normal or ultraviolet-attenuated cercariae of *Schistosoma japonicum* or *S. mansoni*. Parasite Immunol.

[19] Hewitson JP, Hamblin PA, Mountford AP (2005). Immunity induced by the radiation-attenuated schistosome vaccine. Parasite Immunol.

[20] Ratcliffe EC, Wilson RA (1991). The magnitude and kinetics of delayed-type hypersensitivity responses in mice vaccinated with irradiated cercariae of *Schistosoma mansoni*. Parasitol.

[21] Anderson S, Shires VL, Wilson RA, Mountford AP (1998). In the absence of IL-12, the induction of Th1-mediated protective immunity by the attenuated schistosome vaccine is impaired, revealing an alternative pathway with Th2-type characteristics. Eur J Immunol.

[22] Wynn TA, Jankovic D, Hieny S, Cheever AW, Sher A (1995). IL-12 enhances vaccine-induced immunity to *Schistosoma mansoni* in mice and decreases T helper 2 cytokine expression, IgE production, and tissue eosinophilia. J Immunol.

[23] Sato H, Kamiya H (1998). Accelerated influx of dendritic cells into the lymph nodes draining skin sites exposed to attenuated cercariae of *Schistosoma mansoni* in guinea-pigs. Parasite Immunol.

[24] Hewitson JP, Hamblin PA, Mountford AP (2007). In the absence of CD154, administration of interleukin-12 restores Th1 responses but not protective immunity to *Schistosoma mansoni*. Infect Immun.

[25] Amigorena S, Savina A (2010). Intracellular mechanisms of antigen cross presentation in dendritic cells. Curr Opin Immunol.

